# Resistant depression. Clinical manifestations and diagnosis. Purposely a case

**DOI:** 10.1192/j.eurpsy.2023.1788

**Published:** 2023-07-19

**Authors:** S. M. Bañón González, N. Ogando Portilla, O. Sobrino cabra, B. Gamo Bravo, F. García Sánchez

**Affiliations:** 1 Hospital Universitario Infanta Sofia; 2 Hospital Universitario Rey Juan Carlos; 3 Hospital Universitario de Móstoles, Madrid, Spain

## Abstract

**Introduction:**

The term “depression” can be used in different senses: it can be a syndrome, a mood state, a mental disorder, and all of them are distinct clinical conditions…There are no pathognomonic features of bipolar/unipolar depression. A good medical history is the most important component of the evaluation. We have to use clinical variables and differential epidemiology for a correct diagnosis.

**Objectives:**

They both analyze clinical, psychopathological and epidemiological characteristics of resistant depression and they review causes, incidence, prevalence, diagnostic, therapeutic tools and the importance of maintaining the treatment, because the abandonment of the treatment is a good predictor of possible relapses.

**Methods:**

A literature Review of the last five years concerning resistant depression has been done: prevalence, incidence, pathogenesis and its relationship with other psychiatric disorders encoded in DSM-V.

**Results:**

Unipolar major depression (major depressive disorder) is characterized by a history of one or more major depressive episodes and no previous history of mania or hypomania symptoms. A major depressive episode is presented with five or more of the following nine symptoms for at least two consecutive weeks; at least one of them must be either a depressed mood or a loss of interest or pleasure. In addition, the symptoms must cause significant distress or psychosocial impairment, and not be a direct result of a substance or general medical condition.

**Conclusions:**

Symptoms of unipolar depression in adults can overlap with symptoms of other psychiatric and general medical disorders. Unipolar depression needs to be distinguished from these other disorders to prevent inappropriate treatment.

**Disclosure of Interest:**

None Declared

